# Atrial fibrillation activation patterns predict freedom from arrhythmias after catheter ablation: utility of ExTRa mapping™

**DOI:** 10.3389/fcvm.2023.1161691

**Published:** 2023-07-28

**Authors:** Daisetsu Aoyama, Shinsuke Miyazaki, Kanae Hasegawa, Ryohei Nomura, Shota Kakehashi, Moe Mukai, Machiko Miyoshi, Junya Yamaguchi, Yusuke Sato, Yuichiro Shiomi, Hiroyuki Ikeda, Kentaro Ishida, Hiroyasu Uzui, Hiroshi Tada

**Affiliations:** ^1^ Department of Cardiovascular Medicine, Faculty of Medical Sciences, University of Fukui, Fukui, Japan; ^2^ Department of Cardiovascular Medicine, Tokyo Medical and Dental University, Tokyo, Japan

**Keywords:** catheter ablation, atrial fibrillation, activation pattern, heart atria, heart conduction system, rotors

## Abstract

**Background:**

Mechanisms underlying atrial fibrillation (AF) are widely complex and vary tremendously among individuals.

**Objectives:**

This retrospective study aimed to investigate the association between AF activation patterns and clinical outcomes post-ablation.

**Methods:**

Fifty-five AF patients (64.0 ± 12.9 years; 41 men; 17 paroxysmal) underwent bi-atrial endocardial driver mapping during AF pre-ablation with a real-time phase mapping system (ExTRa Mapping). The nonpassively activated ratio (%NP) of meandering rotors and multiple wavelets relative to the recording time was evaluated in 26 atrial segments [15 in the left atrium (LA) and 11 in the right atrium]. Irrespective of the mapping results, all patients underwent standard AF ablation via cryoballoons and/or radiofrequency catheters.

**Results:**

In a median follow-up interval of 27(14–30) months, 69.1% of patients were free from recurrent arrhythmias and antiarrhythmic drugs at one year post-procedure. Patients with recurrent AF were more likely to have non-paroxysmal AF, a significantly larger LA size, and higher LA maximal %NP(LA_max_%NP) and LA anterior wall %NP(LAAW%NP) than those without recurrent AF. A multivariate Cox regression analysis showed that both an LA_max_%NP (hazard ratio [HR] = 1.075; 95% confidence interval [CI] = 1.02–1.14, *p* = 0.012) and LAAW%NP (HR = 1.061; 95% CI = 1.01–1.11, *p* = 0.013) were independent predictors of atrial arrhythmia recurrence. The optimal cutoff points for the LA_max_%NP and LAAW%NP for predicting AF recurrence were 64.5% and 60.0%, respectively. A Kaplan-Meier analysis demonstrated that both an LA_max_%NP > 64.5% (*p* = 0.0062) and LAAW%NP > 60.0% (*p* = 0.014) were associated with more frequent AF recurrences.

**Conclusion:**

Baseline AF activation pattern mapping may aid in predicting freedom from arrhythmias after standard AF ablation procedures.

## Introduction

Atrial fibrillation (AF) has a major impact on global morbidity and mortality rates as the most common of all sustained cardiac arrhythmias worldwide. Currently, catheter ablation is a well-accepted therapeutic strategy for all types of AF, as confirmed by various guidelines (1). However, the sprocedure's success rates are limited, particularly in non-paroxysmal AF (2). Although various adjunctive ablation strategies for eliminating the AF maintenance mechanism have been devised, including left atrial (LA) linear ablation (i.e., ablation of the roof and mitral isthmus line), ablation of complex fractionated atrial electrograms, and magnetic resonance imaging-guided fibrosis ablation, they have not indicated any significant efficacy benefit over pulmonary vein (PV) isolation alone in non-paroxysmal AF patients (3, 4). AF activation flow pattern mapping has been developed as a method to detect action potential sources in the atriums of AF patients (5, 6). This technology, through the use of various devices has the potential to distinguish between active and passive rotational activities. This method can be used to determine which active sources significantly contribute to the perpetuation of fibrillation in the atrium. However, electrical rotor ablation, which identifies and ablates the AF driver as the causative mechanism of AF, has demonstrated a wide variability in its success rates (7–9).

A novel phase mapping system (ExTRa Mapping™, NIHON KOHDEN, Tokyo, Japan), which can visualize intra-atrial signals by means of a specialized artificial intelligence algorithm, was developed to address issues with other mapping systems (6). It provides a high-density movie with a nonpassively activated ratio, which is the ratio of meandering AF rotors and multiple wavelets assumed to contain AF drivers to the recording time. We hypothesized that the results of real-time driver mapping may predict clinical outcomes after standard AF ablation. This study aimed to investigate the association between the baseline AF activation patterns and clinical outcomes after standard AF ablation.

## Methods

### Study population

This was a single-center retrospective cohort analysis. A total of 62 patients completed bi-atrial endocardial driver mapping during AF pre-ablation with a real-time phase-mapping system, ExTRa Mapping, between April 2019 and April 2020. Patients with severe LA dilatation [LA dimension (LAD) > 50 mm] and those with a follow-up period of <1 year were excluded. Additionally, 4 patients taking antiarrhythmic agents (class I, III, and bepridil) at the last follow-up were also excluded to remove the impact of antiarrhythmic agents on the rhythm status and clinical outcomes. Ultimately, 55 patients were included in this study. This study was conducted in accordance with the principles of the Declaration of Helsinki. Written informed consent was obtained from all the patients. The hospital's institutional review board approved the study protocol.

### AF ablation protocol and follow-up

Direct oral anticoagulant use was uninterrupted throughout the periprocedural period, and an activated clotting time of 300–350 s was maintained during the procedure. A 20-electrode catheter (10 in the coronary sinus, 8 in the right atrium [RA], 2 in the superior vena cava [SVC]; BeeAT SAOC; Japan Lifeline Co., Ltd., Tokyo, Japan) was inserted through the right jugular vein. Following a transseptal puncture, bi-atrial endocardial driver mapping was performed using the ExTRa mapping™ system during AF (further procedural details are described later). When the baseline rhythm was sinus rhythm, AF was induced by burst pacing from the coronary sinus without administration of isoproterenol, followed by mapping during AF after at least 15 min of waiting. Subsequently, PV isolation was performed using a contact force-sensing irrigated-tip radiofrequency catheter (SmartTouch Surround Flow, Biosense Webster, Diamond Bar, CA, USA) or a 28 mm fourth-generation cryoballoon (Arctic Front Advance, Medtronic, Minneapolis, MN, USA) guided by the CARTO3 (Biosense Webster) or Rhythmia (Boston Scientific, Natick, MA, USA) three-dimensional electroanatomic mapping system. Additional substrate modification, mainly LA roof area ablation with cryoballoon (10), was performed mainly for patients with a relatively large LA, according to the operator's preference. The construction of an LA voltage map ensued using a 20-pole mapping catheter (PentaRay, Biosense Webster, Irvine, CA, USA) or 64-electrode minibasket mapping catheter (Intellamap Orion, Boston Scientific, Marlborough, MA, USA) during coronary sinus pacing. The low-voltage area (LVA) was delineated based on a bipolar voltage of <0.5 mV. No ablation targeting those LVAs was performed in any patients.

We continued in-hospital electrocardiogram monitoring for 3–5 days after the procedure. Regular follow-up consisted of outpatient clinic visits at 1 and 3 months after the procedure. Subsequent follow-up visits consisted of a clinical interview, 12-lead electrocardiogram, and/or 24-hour Holter electrocardiogram recordings performed every 3 months. Anticoagulation therapy was continued for at least three months. Recurrence was defined as an atrial arrhythmia lasting longer than 30 s after a 3-month blanking period following the latest guidelines (1). Clinical outcomes were examined using electronic medical records.

### Real-time phase mapping analysis

**Figure 1:**
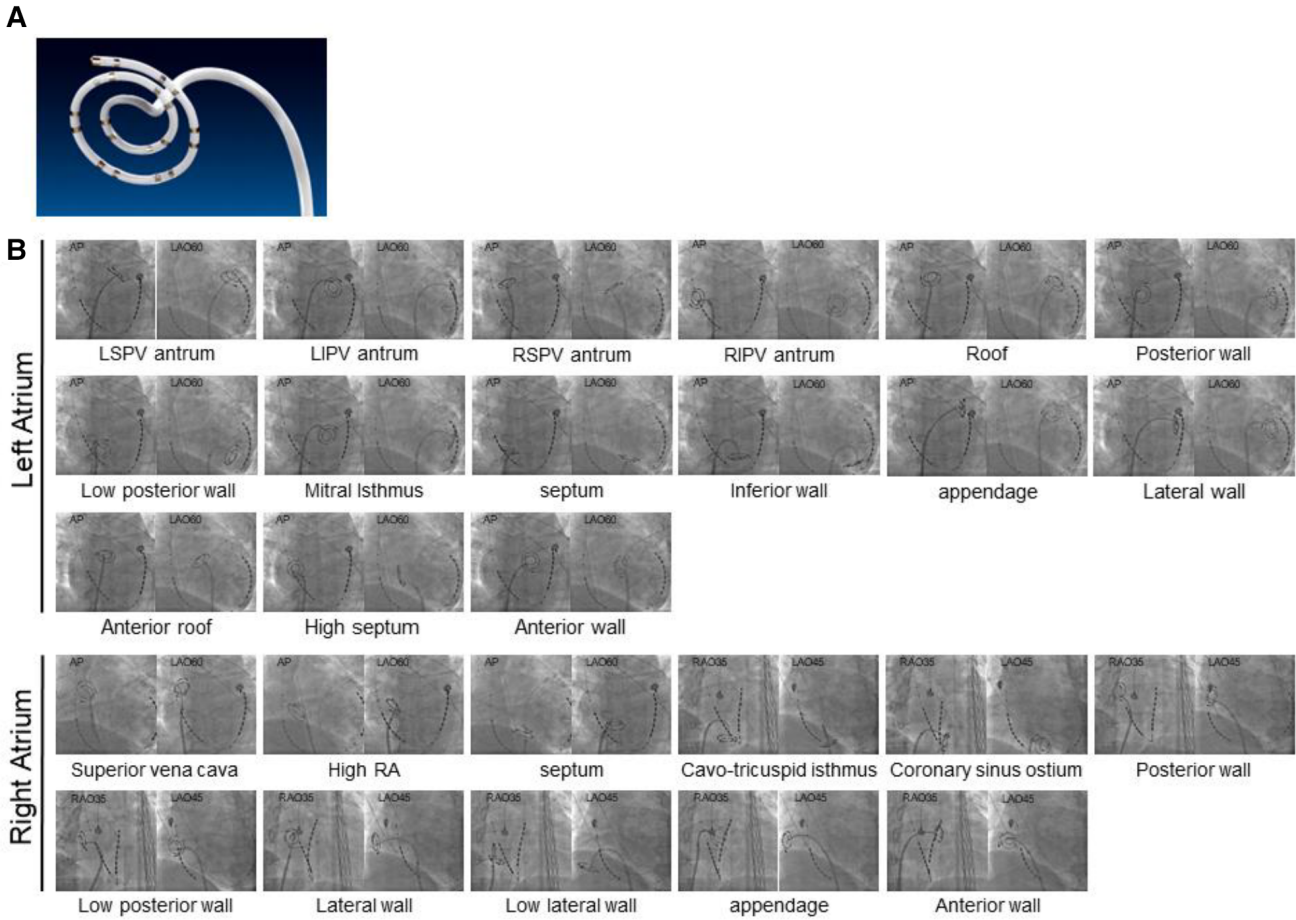
Fluoroscopic images of bi-atrial segments evaluated for activation patterns. (**A**) A deflectable 20-pole spiral-shaped mapping catheter (Reflexion HD, St. Jude Medical, St. Paul, MN, USA). (**B**) Fluoroscopic images of 26 atrial segments (15 LA and 11 RA) evaluated for activation patterns are shown. AP, anteroposterior; LA, left atrial; LAO, left anterior oblique; LI, left inferior; LS, left superior; PV, pulmonary vein; RA, right atrial; RAO, right anterior oblique; RI, right inferior; RS, right superior.

To detect the distribution of AF drivers, a commercially available online real-time phase mapping system (ExTRa Mapping™ system) was used (6). This mapping system was based on 41 bipolar intra-atrial electrograms (20 unipolar, 12 physiological bipolar, and 9 virtual bipolar electrocardiograms) per 5 cm^2^, recorded by a deflectable 20-pole spiral-shaped mapping catheter with a diameter of 2.5 cm (Reflexion HD, St. Jude Medical, St. Paul, MN, USA) (Figure 1A), and the analysis results are automatically calculated. Catheter contact was carefully confirmed through electrogram recording and fluoroscopy. The reliability color was automatically judged from the number of electrocardiograms with an amplitude above 0.03 mV among the 41 electrocardiograms and visualized in real-time on the system. This system can provide a high-density (>4 signals/cm^2^) movie of the contact mapping area in real-time (6).

To determine the location of AF drivers, nonpassively activated areas, in which rotational activations were frequently observed and multiple wavelets were also partly observed, were automatically detected according to the value of the nonpassively activated ratio (%NP) (6). The %NP indicates the ratio of the nonpassively activated period (comprised of meandering rotors and multiple wavelets assumed to contain AF drivers) to the recording time, and the recording time was set to 8 s as a technically maximum setting within a range of 5–8 s. A longer recording time of 8 s, relative to 5 s, improves instability and increases reliability (11). Based on the 8 s wave dynamics during AF, each phase map was automatically created (Supplementary Video S1). The %NP was evaluated in 26 atrial segments to cover all atrial areas: 15 segments were in the LA, consisting of the left superior PV antrum, left inferior PV antrum, right superior PV antrum, right inferior PV antrum, roof, posterior wall, low posterior wall, mitral isthmus, septum, inferior wall, appendage, lateral wall, anterior roof, high septum, and anterior wall; 11 were in the RA, comprised of the SVC, high RA, septum, cavo-tricuspid isthmus, coronary sinus ostium, posterior wall, low posterior wall, lateral wall, low lateral wall, appendage, and anterior wall (Figure 1B). In addition, two sequential repetitive recordings (a total of 16 s) were acquired at each site to obtain higher reliability. The average %NP value was then calculated for each site where sufficient tissue-catheter contact was confirmed. All mapping data were analyzed offline after the procedure. No ablation targeting those high % NP areas was performed in any patients.

### Statistical analysis

Continuous data are expressed as means ± standard deviation for normally distributed variables and as the median [25th, 75th percentiles] for non-normally distributed variables and were compared using Student's *t*-test or Mann-Whitney *U*-test, respectively. Categorical variables were compared using the chi-squared test. A Cox proportional hazard model was used for multivariate analyses to identify independent preprocedural and procedural parameters for recurrent atrial arrhythmias, with the entry criteria of *p* < 0.05 on univariate analysis. Because of the strong correlation between the LA maximal %NP (LA_max_%NP) and LA anterior wall %NP (LAAW%NP), two models were created for each parameter. *Model A* was created using LA_max_%NP and *Model B* was created using LAAW%NP. Receiver operating characteristic (ROC) analysis was used to evaluate the predictive value of LA_max_%NP and LAAW%NP for the determination of the presence or absence of AF recurrence. The area under the curve (AUC) was calculated, and possible cutoff points were selected. Kaplan-Meier analysis was used to evaluate atrial arrhythmia recurrence in patients, and the log-rank test was used to compare groups. A *p*-value of *p* < 0.05 was considered statistically significant. Statistical analyses were performed using JMP version 12.0 (SAS Institute Inc., Cary, NC, USA).

## Results

### Patient characteristics and bi-atrial mapping

**Figure 2:**
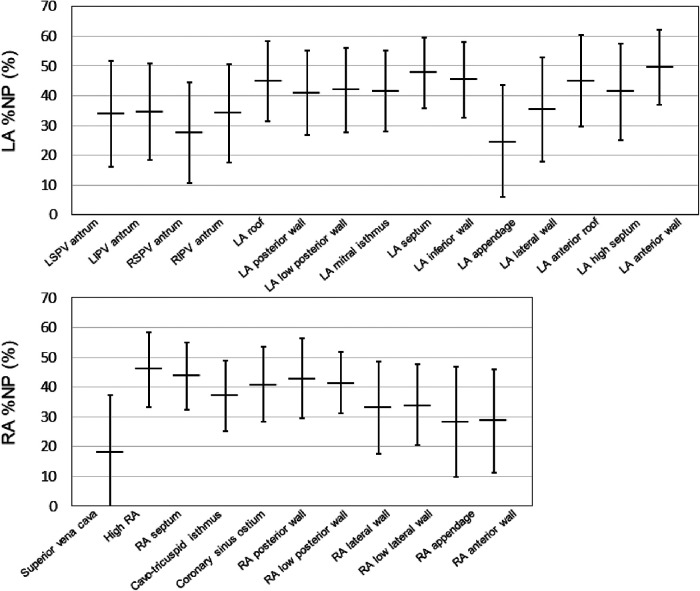
Baseline %NP values for each evaluated bi-atrial segment. The horizontal line indicates the mean %NP values of each of the 26 atrial segments (15 LA and 11 RA) in all the patients. The black line represents the standard deviation for each value. %NP, nonpassively activated ratio; LA, left atrial; LI, left inferior; LS, left superior; PV, pulmonary vein; RA, right atrial; RI, right inferior; RS, right superior.

Fifty-five patients with AF (64.0 ± 12.9 years, 41 men, 17 paroxysmal AF) who underwent bi-atrial endocardial phase mapping followed by AF ablation were included. Among them, 18 (32.7%) patients required AF induction because the baseline rhythm was sinus rhythm. The distribution of the average %NP values for each of the 26 atrial segments (15 LA and 11 RA) is shown in Figure 2. Driver activity was not present throughout the AF mapping period in any of the mapping areas. The maximal and mean %NPs were significantly higher in the LA than in the RA (maximal %NP: 60.5 ± 8.7% vs. 55.0 ± 9.1%, *p* = 0.0003; mean %NP: 38.7 ± 8.0% vs. 35.2 ± 8.7%, *p* = 0.0048). Furthermore, the maximal and mean %NPs in 4 PVs and SVC were significantly lower than those seen in the remaining segments, except for those observed in the LA/RA appendage (bi-atrial body) (maximal %NP; 49.5 ± 12.6 vs. 60.8 ± 7.4, *p* < 0.0001; mean %NP: 29.5 ± 9.3 vs. 41.0 ± 8.2, *p* < 0.0001). Moreover, maximal and mean %NPs in the LA/RA appendage were significantly lower than those in the bi-atrial body (maximal %NP; 36.1 ± 17.0 vs. 60.8 ± 7.4, *p* < 0.0001, mean %NP; 27.0 ± 14.4 vs. 41.0 ± 8.2, *p* < 0.0001). LVAs in the LA were identified in six (10.9%) patients (on the anterior wall in three, on the septum in two, and on the posterior wall in one). The median %LVA (LVA/total LA surface area) was 3.3% (0.9%–6.5%) and the value was less than 10% in all patients.

### Clinical outcomes and predictors of arrhythmia recurrence

All patients underwent a successful PV isolation, and an LA roof line ablation was performed in 42 patients (76.4%). A cavo-tricuspid isthmus linear ablation, SVC isolation, bottom line ablation, and ablation of non-PV foci were added in 52, 7, 6, and 2 patients, respectively (Table 1). No ablation targeting high %NP areas or low voltage areas was performed in any patients. No complications were observed except for in one patient who experienced transient gastric hypomotility and another with cardiac tamponade. AF was terminated during or after ablation in 9 patients (paroxysmal AF in 7 and persistent AF in 2), while electrical cardioversion was required to restore sinus rhythm in the remaining 46 patients. Of the 9 patients, AF terminated during and immediately after the PV isolation in 4 patients (left superior PV in 3 and right inferior PV in 1) and during and soon after the roof line ablation in the remaining 5 patients. The mean %NP value of the area where AF was terminated by ablation was 39.9%, and the area was not the highest %NP area in all 9 patients except for in one patient. The %NP values were similar in the patients with AF termination by ablation and those by electrical cardioversion (maximal %NP; 60.4 ± 6.9 vs. 60.5 ± 9.1, *p* = 0.99, mean %NP; 36.3 ± 7.1 vs. 39.2 ± 8.1, *p* = 0.32). During a median follow-up interval of 27 (14–30) months, 69.1% of the patients were noted to be free from recurrent arrhythmias and antiarrhythmic drug use. No hospitalizations due to cardiovascular events were recorded; however, one patient died due to acute lymphoblastic leukemia, 2 years after the procedure.

**Table 1:** Patient clinical characteristics and ablation strategy.

	Overall (*n* = 55)	With AF recurrence (*n* = 17)	Without AF recurrence (*n* = 38)	*p-*value
Age, years	64.0 ± 12.9	59.1 ± 13.8	66.2 ± 12.0	0.070
Male gender, *n* (%)	41 (74.5)	15 (88.2)	26 (68.4)	0.20
Body mass index (kg/m^2^)	24.1 ± 3.8	25.3 ± 3.5	23.6 ± 3.8	0.097
Chronic heart failure, *n* (%)	5 (4.5)	1 (5.9)	4 (10.5)	0.44
Hypertension, *n* (%)	26 (47.3)	7 (41.2)	19 (50.0)	0.59
Diabetes mellitus, *n* (%)	2 (3.6)	0 (0.0)	2 (5.3)	0.17
Old cerebral infarction, *n* (%)	3 (5.5)	1 (5.9)	2 (5.3)	0.93
Vascular disease, *n* (%)	1 (1.8)	0 (0.0)	1 (2.6)	0.36
CHADS_2_ score	0.9 ± 1.0	0.7 ± 1.0	1.0 ± 1.0	0.19
CHA_2_DS_2_-VASc score	1.8 ± 1.5	1.2 ± 1.4	2.0 ± 1.5	0.052
Non-paroxysmal AF, *n* (%)	38 (69.1)	15 (88.2)	23 (60.5)	0.041
AADs resistance, *n* (%)	11 (20.0)	2 (11.8)	9 (23.7)	0.29
Left ventricular ejection fraction, %	60.9 ± 8.0	60.8 ± 8.8	60.9 ± 7.7	0.87
Left atrial dimension, mm	40.0 ± 5.4	42.1 ± 4.0	39.1 ± 5.7	0.038
Left atrial volume index, mm/m^2^	47.8 ± 4.1	47.4 ± 3.7	48.0 ± 4.3	0.73
LA low-voltage areas, *n* (%)	6 (10.9)	1 (5.9)	5 (13.9)	0.37
Ablation strategy				
Cavo-tricuspid isthmus line, *n* (%)	52 (94.5)	16 (94.1)	36 (94.7)	0.80
Superior vena cava isolation, *n* (%)	7 (12.7)	2 (11.8)	5 (13.2)	0.98
Mitral isthmus line, *n* (%)	0 (0.0)	0 (0.0)	0 (0.0)	
Roof line, *n* (%)	42 (76.4)	12 (70.6)	30 (78.9)	0.48
Bottom line, *n* (%)	6 (10.9)	4 (23.5)	2 (5.3)	0.14
Low voltage area, *n* (%)	0 (0.0)	0 (0.0)	0 (0.0)	
Non-pulmonary vein foci, *n* (%)	2 (3.6)	2 (11.8)	0 (0.0)	0.11
Rotor ablation, *n* (%)	0 (0.0)	0 (0.0)	0 (0.0)	

Values are reported as the mean ± standard deviation or number of patients (%), unless otherwise noted. AF, atrial fibrillation; AADs, antiarrhythmic drugs.

**Figure 3:**
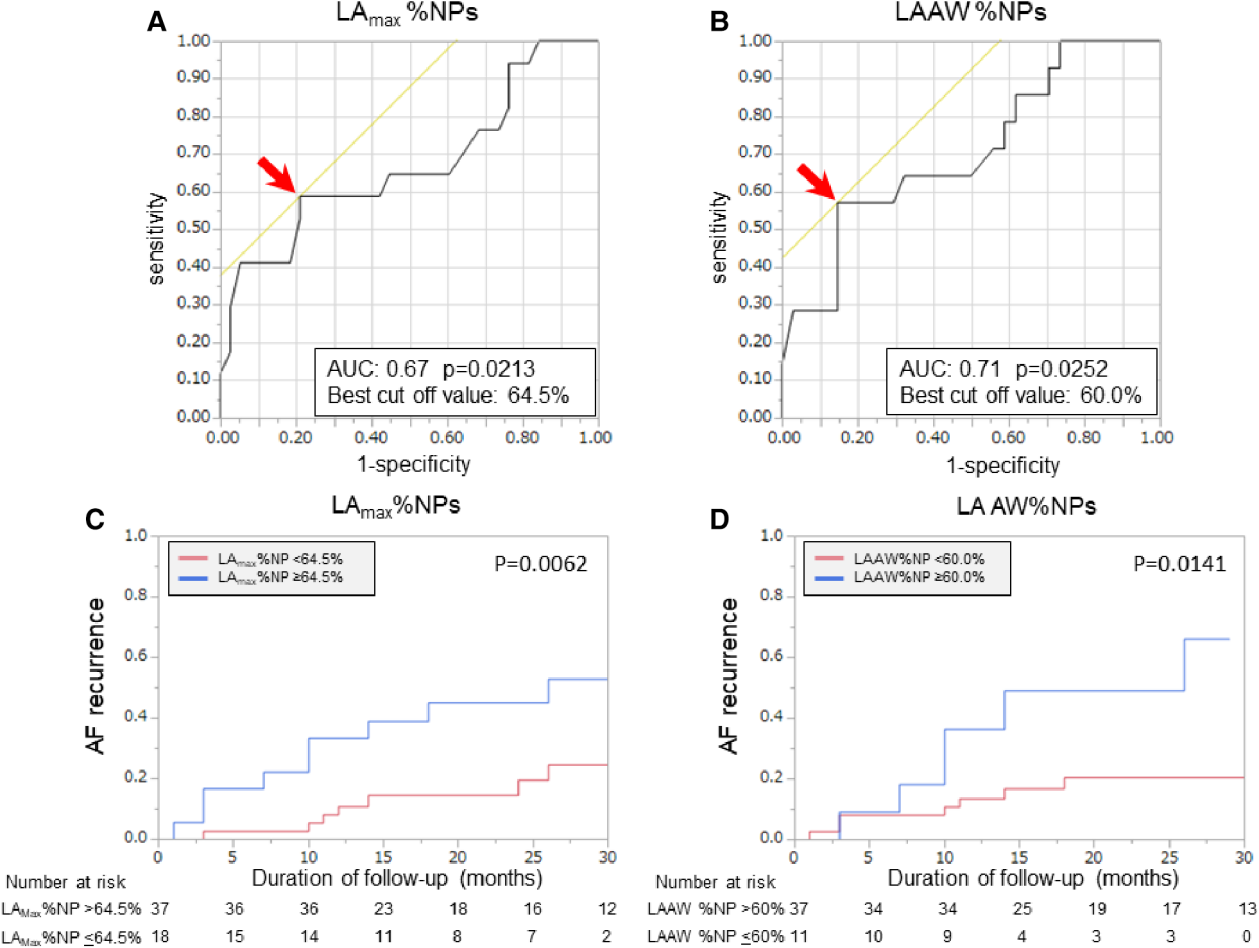
Baseline %NP values and clinical outcomes after the procedure. Receiver operating characteristic curves show the optimal cut-off values (red arrows) of LA_max_%NP (**A**) and LAAW%NP (**B**) for predicting freedom from arrhythmia after AF ablation. In the Kaplan-Meier analysis, LA_max_%NP >64.0% (**C**) and LAAW%NP >60.0% (**D**) were significantly associated with higher arrhythmia recurrence after the procedure. AF, atrial fibrillation; AUC, area under the curve; LAAW%NP, left atrial anterior wall nonpassively activated ratio; LA_max_%NP. Left atrial maximal nonpassively activated ratio.

Patients with recurrent AF were more likely to have non-paroxysmal AF (*p* = 0.041) and a significantly larger LAD (*p* = 0.038) than patients without recurrent AF, but there was no significant difference in the ablation strategies between the patients with and those without recurrent AF (Table 1). Additionally, the LA_max_%NP value (*p* = 0.015) and LAAW%NP value (*p* = 0.022) were significantly higher in patients with AF recurrence than those without, although there were no between-group significant differences in the RA %NP values (Table 2). LAAW%NP correlated significantly with LA_max_%NP with moderate accuracy (*r* = 0.66, *p* < 0.001), and LAAW%NP had the strongest correlation with LA_max_%NP among all the LA %NP values. In multivariate Cox proportional hazard model analysis, LA_max_%NP (hazard ratio [HR] = 1.075; 95% confidence interval [CI] = 1.02–1.14, *p* = 0.012) was the only independent predictor of AF recurrence after the procedure (Table 3, *Model A*). LAAW%NP [odds ratio (OR) = 1.061; 95%CI = 1.01–1.11; *p* = 0.013] also predicted arrhythmia recurrence after the procedure (Table 3, *Model B*). An ROC analysis demonstrated that an LA_max_%NP value of 64.0% was the optimal cutoff value for predicting AF recurrence with 58.8% sensitivity, 79.0% specificity, 55.6% positive predictive value, and 81.1% negative predictive value (AUC:0.67, *p* = 0.021; Figure 3A). Similarly, an LAAW %NP value of 60.0% was the optimal cutoff value for predicting AF recurrence with 57.1% sensitivity, 85.3% specificity, 61.5% positive predictive value, and 82.9% negative predictive value (AUC:0.71, *p* = 0.025; Figure 3B). Kaplan-Meier analysis showed that LA_max_%NP values ≤64.0% (log-rank, *p* = 0.0062) and LAAW %NP values ≤60.0% (log-rank, *p* = 0.014) were associated with significantly higher freedom from arrhythmia after the procedure (Figure 3C,D).

**Table 2:** Procedural characteristics.

	No. with available data	With AF recurrence	Without AF recurrence	*p-*value
%NP values in LA				
LA max %NP, %	55	64.7 ± 8.8	58.6 ± 8.1	0.015
LA mean %NP, %	55	40.8 ± 4.0	37.9 ± 6.0	0.26
Left superior PV antrum %NP, %	54	39.7 ± 18.4	31.4 ± 17.2	0.18
Left inferior PV antrum %NP, %	50	40.9 ± 18.5	32.0 ± 14.8	0.061
Right superior PV antrum %NP, %	52	22.8 ± 15.0	30.0 ± 17.3	0.25
Right inferior PV antrum %NP, %	50	37.0 ± 15.3	32.7 ± 17.0	0.36
LA roof %NP, %	48	46.5 ± 12.6	44.3 ± 13.9	0.90
LA posterior wall %NP, %	49	46.4 ± 10.8	38.4 ± 15.0	0.15
LA low posterior wall %NP, %	45	43.9 ± 11.5	41.3 ± 15.1	0.86
LA mitral isthmus %NP, %	39	45.0 ± 13.5	40.4 ± 13.5	0.32
LA septum %NP, %	50	49.2 ± 14.2	46.9 ± 10.8	0.51
LA inferior wall %NP, %	44	44.5 ± 16.3	45.5 ± 11.5	0.92
LA appendage %NP, %	53	23.6 ± 20.4	25.1 ± 18.3	0.50
LA lateral wall %NP, %	48	40.9 ± 16.6	33.2 ± 17.7	0.19
LA anterior roof %NP, %	46	45.9 ± 14.6	44.6 ± 15.9	0.87
LA high septum %NP, %	49	38.4 ± 21.1	42.5 ± 14.2	0.37
LA anterior wall % NP, %	48	56.3 ± 11.4	46.9 ± 12.0	0.022
%NP values in RA				
RA max %NP, %	55	53.3 ± 8.8	55.8 ± 9.2	0.34
RA mean %NP, %	55	32.8 ± 8.1	36.3 ± 8.9	0.16
Superior vena cava %NP, %	51	16.6 ± 20.7	18.7 ± 18.7	0.35
High RA %NP, %	40	42.6 ± 14.1	47.6 ± 11.7	0.24
RA septum, %NP, %	43	44.5 ± 11.5	43.3 ± 11.3	0.68
Cavo-tricuspid isthmus %NP, %	32	35.4 ± 13.8	37.8 ± 11.3	0.62
Coronary sinus ostium %NP, %	34	40.3 ± 11.2	41.3 ± 13.3	0.71
RA posterior wall %NP, %	46	39.2 ± 16.8	44.4 ± 11.8	0.20
RA low posterior wall %NP, %	32	43.7 ± 9.9	40.5 ± 10.4	0.37
RA lateral wall %NP, %	49	33.8 ± 18.6	32.7 ± 14.2	0.80
RA low lateral wall %NP, %	45	32.8 ± 13.2	34.7 ± 13.8	0.50
RA appendage %NP, %	54	21.9 ± 11.9	31.2 ± 20.2	0.051
RA anterior wall %NP, %	36	28.4 ± 16.8	28.9 ± 18.0	0.80

Values are reported as the number of patients (%), unless otherwise noted.

%NP, non-passively activated ratio; LA, left atrium; PV, pulmonary vein; RA, right atrium.

**Table 3:** Predictors of AF recurrence in the multivariate analysis of a Cox proportional hazard model.

	Hazard ratio (95% confidence interval)	*p*-value
Model A		
LA max %NP, %	1.075 (1.016–1.143)	0.012
Persistent AF, *n*	2.341 (0.555–16.262)	0.27
LA dimension, mm	1.082 (0.971–1.211)	0.15
Model B		
LA anterior wall %NP, %	1.061 (1.012–1.118)	0.013
Persistent AF, *n*	2.077 (0.496–14.285)	0.34
LA dimension, mm	1.087 (0.967–1.232)	0.16

%NP, non-passively activated ratio; AF, atrial fibrillation; LA, left atrial.

## Discussion

The present study demonstrated the results of bi-atrial endocardial driver mapping using a real-time phase-mapping system prior to AF ablation. Our results can be summarized as follows: (1) driver activity was not present throughout the AF mapping period and most of them were transitory; (2) drivers were most frequently observed at the LA anterior wall, LA septum, and LA roof area; (3) passive activity was dominant in the 4 PVs, SVC, RA/LA appendages, and lateral LA during most of the mapping time; and (4) the majority of the patients were arrhythmia-free after standard AF ablation even without targeting the highest driver activity area. However, baseline LA driver activity level predicted arrhythmia freedom after ablation.

### AF mechanisms and driver mapping

Electric and structural remodeling is fundamental to the AF disease process, and the appearance of fibrosis increases substrate dimensions. This remodeling process is thought to lead to rotor facilitation and the multiplication of randomly circulating waves associated with a decreased atrial refractory period and heterogeneous tissue structure (11). Multiple atrial wavelets, meandering rotors, macroreentries, and localized (focal or reentrant) sources have all been reported to contribute to the substrate of persistent AF (12, 13). In humans, AF drivers have been demonstrated during endocardial and epicardial mapping with the use of multielectrode tools or noninvasive torso electrode arrays using activation, spectral, or phase mapping (14–16), although the mapping results are inconsistent, presumably due to the different mapping methodologies involved.

The ExTRa mapping system is a commercially available mapping system that enables real-time phase-mapping during AF ablation. The system demonstrates rotational activations (meandering rotors and/or multiple wavelets) and passively activated planar wave propagation with high spatial density (6). The reliability of the phase mapping data obtained by the system (the threshold setting was 0.03 mV) has already been validated through the simultaneous measurement of high-resolution optical membrane potential mapping in rabbit ventricular myocardium (17), and the system has been used in some clinical studies for human AF (17, 18). The advantage of the system lies in its capability for real-time phase mapping based on the local electrograms recorded by contact mapping. One limitation of the system is its difficulty in identifying intermittent firing and spatial meandering because of sequential temporospatial mapping, as in other systems. This study is distinctive from previous research that employed the ExTRa mapping system (18, 19) because this study not only mapped the LA but also the RA to identify the AF driver domain, unlike previous studies that only mapped the LA. This study also utilized a considerably longer recording time (sequential repetitive 8 s recording) to improve the temporal stability unlike the single 5 s recordings used in previous studies (20).

### Real-time phase mapping of human AF

The present phase mapping data showed unstable reentries in wide spatial domains, with short-lived generated rotors, mixed with other mechanistic patterns of activation, in accordance with the results of panoramic noninvasive mapping data (15). Our bi-atrial contact mapping also observed the anatomic distribution of AF drivers and identified predominant domains such as the LA anterior wall, LA septum, and LA roof area. In contrast, passive waves (organized activity) prevailed in the PVs, SVC, LA/RA appendage, and lateral LA throughout the cumulative AF mapping period. Nevertheless, the majority of patients were free from AF after standard AF ablation without targeting specific AF driver areas, although the present study population consisted of patients with a relatively healthy atrium (without significant LVAs). Given these findings, it seems reasonable to consider that 4 PVs and SVC mainly play a role in triggering AF and the atrial body plays a role in driving AF, although the limited mapping period reflects the limitations of sequential endocardial contact mapping.

The current study observed that the presence of high %NP areas was associated with arrhythmia recurrence after AF ablation, indicating that the residual AF substrate might be predisposed to AF recurrence. Nakamura et al. previously showed that nonpassive activations were mainly located in tissue exhibiting heterogenous myocardial fibrosis amongst healthy tissue, which could be detected as heterogenous late gadolinium enhancement on cardiac magnetic resonance imaging (20). Given the study results, we assume that the fibrotic process may have contributed to the development of high %NP areas. This mapping system possesses the utility of identifying the dominant AF driver domain and aids in predicting clinical outcomes after standard AF ablation procedures.

### Ablation targeting AF drivers

Several studies have reported an ablation strategy targeting AF drivers though the results are conflicting (8). If a discrete number of rotors, temporally stable for hours, are present in a limited special domain, ablation targeting the rotors seems to be reasonable (16). However, the periodic occurrence of unstable reentries is theoretically less amenable to ablation. Proposed ablation strategies involve ablation targeting the region with the highest driver activity identified by body surface electrode mapping until AF termination (15), and ablation targeting the area with the highest %NP values identified by the ExTRa mapping system until the reduction of %NP at the area (6). However, randomized prospective studies showing the additional benefit of rotor ablation beyond standard AF ablation are nonexistent, and no definitive mapping technique or ablation endpoint has ever been established. Further studies are needed to characterize the respective contributions of AF drivers and establish appropriate ablation strategies for these drivers.

### Clinical implications

The mechanisms of sustained AF and strategies targeting AF substrates in patients with persistent AF remain unclear. Currently, it is well known that patients with LVAs and advanced atrial disease have worse outcomes than those without LVAs (21). However, limited data have focused on the factors predicting clinical outcomes after AF ablation in patients with AF without advanced atrial disease. The present study included these populations. A real-time phase mapping system could identify the AF driver domain and aid in predicting clinical outcomes after the procedure in these populations. Moreover, this may facilitate the identification of patients who require substrate modification following the standard AF ablation procedure.

### Limitations

First, this was a single-center retrospective observational study, and the study population was relatively small. Second, patients in whom AF did not persist over a 15 min waiting time or terminated during mapping were not included. Third, patients with severe LA dilatation (>50 mm) were not included because good tissue contact with the specific mapping catheter was challenging in patients with a large LA and the accuracy of the driver mapping has not been validated in scar areas, where the signal amplitude is below 0.03 mV. Fourth, the mapping time of AF was limited as with the other mapping systems. Fifth, the impact of AF driver ablation was undetermined because ablation targeting the driver area was not performed in this study.

## Conclusions

Bi-atrial sequential endocardial phase mapping of human AF demonstrated that most AF driver activities were transitory and widely distributed. The baseline AF driver activity level predicted freedom from arrhythmias after a standard AF ablation procedure.

## Data Availability

The raw data supporting the conclusions of this article will be made available by the authors, without undue reservation.

## Supplementary material

SUPPLEMENTARY VIDEO S1

Representative movie of a non-passively activated area. Representative images of a high %NP area on the LA anterior wall. The recording time was set to 8 s and the video is played at 0.1× speed. The value of the %NP and passively activated ratio (%P) was 70% and 30%, respectively. The %NP and %P are listed at the top of the screen, and the %NP also lists the %values for rotors as R and multiple wavelets as M; when rotors, multiple wavelets, and a passively activated pattern appears, the respective letters are highlighted.
